# Gender-Patterns in Cyberbullying Involvement Categories: Insights into the Cyberbully/Victims Subgroup

**DOI:** 10.3390/bs16081303

**Published:** 2026-07-31

**Authors:** Anna Sorrentino, Margherita Santamato, Antonio Aquino

**Affiliations:** 1 Department of Psychology, University of Campania “Luigi Vanvitelli”, 81100 Caserta, Italy; margherita.santamato@unicampania.it; 2 Department of Health Sciences, Magna-Graecia University, Viale Europa, 88100 Catanzaro, Italy; antonio.aquino@unicz.it

**Keywords:** cyberbullying, cyberbully/victims, cybervictimization, gender differences, risk factors

## Abstract

Background: Cyberbullying is a critical public health issue affecting adolescent well-being. This study adopted a socio-ecological framework to examine individual, family, and school-level risk factors associated with three cyberbullying roles—Cyberbullies, Cybervictims, and Cyberbully/Victims—with particular attention to the often-overlooked Cyberbully/Victim subgroup and gender pathways. Methods: A total of 4543 adolescents (Mage = 13.97 years; 52.5% girls) completed the Tabby Improved Checklist. Multinomial logistic regression analyses were conducted separately for boys and girls to identify predictors of involvement in the three cyberbullying roles, including empathy dimensions, moral disengagement, antisocial behavior, aggression toward teachers, and social support, using non-involved adolescents as the reference group. Results: Cyberbully/Victims represented a high-risk profile characterized by elevated moral disengagement, antisocial behavior, and aggression toward teachers, irrespective of gender. Gender-specific patterns also emerged: male Cyberbullies exhibited high cognitive empathy and low affective empathy, whereas female Cybervictims and Cyberbully/Victims reported higher levels of affective empathy. Furthermore, low parental support and monitoring significantly predicted involvement among male Cyberbullies but not among females. Aggression toward teachers emerged as a common risk factor across all cyberbullying roles. Conclusions: These findings underscore the complexity of the Cyberbully/Victim role and support the need for gender-sensitive prevention strategies. Effective prevention efforts should adopt a multi-level approach addressing maladaptive cognitive processes, emotional regulation, and student–teacher relationships to reduce online aggression.

## 1. Introduction

The increased diffusion and availability of the Internet and new communication technologies among people (i.e., smartphones, personal computers, and social networks) enriched their social lives by allowing them to interact with acquaintances and friends, or make new ones (Timeo et al., 2020; Haddock et al., 2022).

However, alongside these benefits (Haddock et al., 2022), one of the most significant risks faced by social media users, particularly adolescents, is cyberbullying (Giumetti & Kowalski, 2022; Sarangi et al., 2022; Craig et al., 2020). Cyberbullying should be defined as any aggressive or bullying-like behaviour directed at an individual or a group through the exclusive use of electronic media (Kasturiratna et al., 2025, p. 102).

Over the past 20 years, cyberbullying has attracted growing interest from the scientific community and is increasingly discussed as a relevant public health concern affecting adolescents on a global scale (Machado et al., 2022). Despite current evidence supporting a tripartite conceptualization of involvement, consisting of cyberbullies, cybervictims, and cyberbully/victims (Zhu et al., 2021), the majority of empirical studies investigating risk factors for cyberbullying involvement focused only on cyberbullies and cybervictims categories of involvement, thus forgetting the cyberbully/victim subgroup.

Identifying risk factors for the involvement in the cyberbully/victim role could be crucial both in terms of designing primary prevention programs and in intervention activities also considering the negative health and psychological effects affecting this particular sub-group, which presents a complex combination of internalised and externalised difficulties, such as emotional dysregulation (Tintori et al., 2025; Quintana-Orts et al., 2024; Kennedy, 2021; A. C. Baldry et al., 2019a), higher levels of engagement in deviant and high-risk behaviours, and interpersonal violence (Azami & Taremian, 2021; Graham & Wood, 2019; Nasaescu et al., 2020).

Cyberbullying is increasingly recognized as a multifactorial phenomenon resulting from the interplay between individual characteristics and the social environments in which adolescents develop. Consequently, explanatory models focusing exclusively on either personal or contextual factors provide only a partial understanding of adolescents’ involvement in cyberbullying. Within this perspective, Bronfenbrenner’s (1979) socio-ecological model offers a comprehensive theoretical framework by conceptualizing adolescent behaviour as the product of continuous interactions between the individual and multiple interconnected environmental systems.

The model posits that individual development is shaped by the dynamic and reciprocal interaction between the developing person and the multiple, interconnected environmental systems with which they interact, thereby enabling the identification of significant risk or protective factors for cyberbullying by situating them within their respective ecological systems. Bronfenbrenner’s socio-ecological model conceptualizes adolescent development as the result of interactions across multiple ecological systems, including the ontogenetic, microsystem, mesosystem, exosystem, and macrosystem levels. Although all these systems contribute to adolescent development, the present study specifically focuses on the ontogenetic and microsystem levels because they represent the most proximal developmental contexts and have been most consistently associated with adolescents’ involvement in cyberbullying. A growing body of research has demonstrated that ontogenetic-level factors, such as empathy and moral disengagement, interact with microsystem-level influences, including parental support, parental monitoring, teacher support, and the broader school environment, in shaping the likelihood of involvement in cyberbullying (Kowalski et al., 2014; Zych et al., 2019a; Sorrentino et al., 2023; Quintana-Orts et al., 2025).

Following the socio-ecological framework, the literature is reviewed according to the ecological level at which each factor primarily operates. We first examine ontogenetic risk factors, followed by family- and school-level influences within the microsystem, thereby providing a theoretically organized overview of the psychosocial determinants of cyberbullying involvement.

### 1.1. Ontogenetic-Level Risk Factors

According to the socio-ecological framework, ontogenetic-level characteristics constitute the most proximal influences on adolescents’ behaviour. Individual socio-emotional competencies and behavioural dispositions may increase or reduce the likelihood of involvement in cyberbullying by shaping how adolescents perceive social situations, regulate their emotions and behaviour, and respond to interpersonal conflict. Empirical evidence has consistently identified empathy (Zych et al., 2019b), moral disengagement (Pornari & Wood, 2010), and antisocial behaviour (Holfeld & Leadbeater, 2015) as key ontogenetic-level factors associated with cyberbullying involvement. Accordingly, the following sections review the evidence concerning these three individual-level characteristics.

*Empathy*. Empathy is considered a multidimensional construct, comprising cognitive (understanding others’ emotions) and affective (sharing others’ feelings) components (Davis, 1983; Hoffman, 1996).

Despite extensive attention to empathy as a potential correlate of adolescents’ involvement in cyberbullying (Zych et al., 2019b), empirical evidence remains inconsistent.

Deficits in both dimensions have been linked to increased cyberbullying (Arató et al., 2022; Ang & Goh, 2010; Zych et al., 2019b), while, conversely, high levels of affective empathy have also been identified as longitudinal risk factors for cyberbullying (Sorrentino et al., 2023). Regarding cybervictimization, several studies have found higher levels of empathy among victims (Arató et al., 2022; Shannen et al., 2021).

Nevertheless, the empathic profiles of cyberbully/victims remain largely unexplored, particularly regarding the relationship between empathy and gender differences in youth involvement in different cyberbullying categories (Zych et al., 2019a).

*Moral disengagement*. The literature shows that cyberbullies often use strategies such as shifting responsibility or dehumanizing the victim to mitigate feelings of guilt (Pornari & Wood, 2010). Additionally, other studies reveal that cybervictims may resort to moral disengagement as a coping mechanism to handle the psychological distress that comes from being targeted (Chen et al., 2017).

Evidence regarding cyberbully/victims remains limited, with some findings suggesting that cyberbully/victims may also display high levels of moral disengagement (Marín-López et al., 2020).

*Antisocial behaviors*. Empirical and longitudinal evidence shows that externalized problematic behaviours, such as antisocial and transgressive behaviours, are strong longitudinal predictors of involvement in cyberbullying. Specifically, antisocial behaviours are significantly associated with both cyberbullying (Holfeld & Leadbeater, 2015; Kim et al., 2017; Sticca et al., 2013; You & Lim, 2016) and cybervictimization (Gámez-Guadix et al., 2013; Holfeld & Leadbeater, 2015; Korchmaros et al., 2014; Modecki et al., 2013). Convergent results also emerge from cross-sectional research: Ybarra and Mitchell (2004) report that cyberbullies exhibit high levels of rule-breaking behaviour (e.g., vandalism, alcohol consumption), while Garaigordobil (2017) finds that both cyberbullies and cybervictims score higher on antisocial behaviour. Overall, review studies further support this model, demonstrating that externalizing and rule-breaking behaviours are significant longitudinal risk factors for both cyberbullying and cybervictimization (Camerini et al., 2020). Despite this body of evidence, the literature on the specific subgroup of cyberbully/victims and their antisocial behaviours remains limited. To our knowledge, there are no studies on this topic; only one study has shown that cyberbully/victims are more vulnerable than both cyberbullies and cybervictims, exhibiting higher levels of aggression and lower levels of self-control (Bayraktar et al., 2015), without directly assessing antisocial behaviour. Furthermore, gender differences within these processes remain largely unexplored.

### 1.2. Microsystem

#### 1.2.1. Family-Level Risk Factors

Within the socio-ecological framework, the family represents one of the primary microsystems influencing adolescent development. Parenting practices and supportive family relationships may either buffer or exacerbate adolescents’ vulnerability to cyberbullying involvement. Empirical evidence has highlighted the role of parental online monitoring (Zhu et al., 2021; Elsaesser et al., 2017) and parental support (López-Castro & Priegue, 2019; Kowalski et al., 2014) in shaping adolescents’ risk of involvement, with conflicting results; accordingly, this section reviews evidence concerning these two family-level factors.

*Parental online monitoring.* Empirical findings concerning the protective or the risk role of parental online monitoring have been mixed, with some studies consistently indicating that parental monitoring exerts a protective effect against both cyberbullying and cybervictimization (Zhu et al., 2021; Elsaesser et al., 2017; Elboj-Saso et al., 2024), and some others suggesting that it may represent a risk factor for youth involvement in cyberbullying (Gómez et al., 2017; Hood & Duffy, 2018). Notably, as far as we know, no studies have examined adolescents who are involved as cyberbully/victims in relation to parental online monitoring. In relation to gender differences, Wright (2017) found that the association between restrictive parental monitoring and cybervictimization was stronger for girls than for boys. Similarly, A. C. Baldry et al. (2019b) reported that a lack of parental online monitoring in male adolescents was associated with a higher likelihood of engaging in cyberbullying, whereas girls experiencing cybervictimization reported higher levels of parental online supervision.

*Parental support*. Adolescents who perceive low levels of support from their parents are consistently more likely to experience cyber victimization (López-Castro & Priegue, 2019; Kowalski et al., 2014; Martins et al., 2016) and to be involved in cyberbullying (Ateş et al., 2018; Doty et al., 2018; López-Castro & Priegue, 2019; Kowalski et al., 2014). The literature examining perceived parental support in different cyberbullying roles is more limited, but available evidence indicates that cyberbully/victims represent the most problematic profile, reporting more family conflicts, weaker parent-child bonds, and lower levels of family cohesion and expressiveness compared to other roles of involvement (Bayraktar et al., 2015; Kokkinos et al., 2016; Buelga et al., 2017). More recent findings also show that high family support exerts a significant protective effect on all three categories of involvement in cyberbullying, including cyberbully/victims (Malinowska-Cieślik et al., 2022). Research on gender differences in these associations is still limited. However, current moderation analyses suggest that parental support may be more protective for girls. Girls typically report higher levels of perceived parental support, which is linked to a lower likelihood of experiencing cybervictimization and, in some cases, cyberbullying. In contrast, these associations appear weaker for boys (López-Castro & Priegue, 2019). Consistent with this pattern, Piazuelo-Rodríguez et al. (2024) report that parental support exerts a direct regulatory effect on both cybervictimization and cyberbullying among adolescents, with a notably more substantial impact among girls. In contrast, the protective influence is substantially less pronounced among boys.

#### 1.2.2. School-Level Risk Factor

The school constitutes another central developmental context within the socio-ecological model. Relationships with teachers and peers contribute substantially to adolescents’ social adjustment and may represent important protective or risk factors for involvement in cyberbullying. Research has shown that conflictual student–teacher relationships (Longobardi et al., 2018; D. Marengo et al., 2018) and low peer support (A. C. Baldry et al., 2015; Kowalski et al., 2014) are associated with higher risk; accordingly, the following sections examine aggression toward teachers and peer support.

*Aggression toward teachers.* The extant literature acknowledges the pivotal function of the quality of the student-teacher relationship in predicting victimization and aggression among adolescents. Conflictual, distant or, unsupportive relationships have been shown to heighten the risk in students who are already isolated or rejected, and to encourage aggression towards peers (Longobardi et al., 2018; D. Marengo et al., 2018, 2021; Elledge et al., 2016; Wang et al., 2015; Hughes & Im, 2016). Moreover, some evidence suggests that online insults, teasing, and physical aggression towards teachers are more prevalent among male than female students (Sorrentino & Farrington, 2019). However, no study has yet tested whether aggression towards teachers constitutes a risk factor for involvement in the full spectrum of cyberbullying roles (cyberbullies, cybervictims, cyberbully/victims), nor whether these associations vary by gender. Findings from school bullying research offer a crucial theoretical reference: conflictual teacher–student relationships increase the risk of both perpetration (Hughes & Im, 2016; Longobardi et al., 2018; Wang et al., 2015) and victimization (Elledge et al., 2016; Longobardi et al., 2018; D. Marengo et al., 2018). Victim–aggressors typically show hybrid profiles combining vulnerabilities and aggressive tendencies (Quintana-Orts et al., 2024), with girls appearing especially likely to assume this dual role under conditions of high teacher–student conflict (D. Marengo et al., 2018). In this regard, Ortega-Barón et al. (2017) found that individuals who perpetrate cyberbullying on a severe and occasional basis exhibit a pronounced rejection of school authority, and recent longitudinal evidence shows that negative student–teacher relationships predict increased cyberbullying perpetration over time (Thornberg et al., 2025). However, no studies to date have systematically examined how violence against teachers relates to the full spectrum of cyberbullying involvement (cyberbully, cybervictim, cyberbully/victim), nor have they accounted for potential gender differences.

*Peer support.* In addition to family support, peer support consistently emerges as a key protective factor against involvement in both cyberbullying and cybervictimization (N. Marengo et al., 2021). Higher levels of perceived peer support are associated with a lower likelihood of engaging in cyberbullying or becoming cybervictims (N. Marengo et al., 2021). Conversely, limited or poor peer support has been identified as a significant risk condition, with some evidence suggesting that it is associated with youth involvement in cyberbullying (A. C. Baldry et al., 2015) and cybervictimization (A. C. Baldry et al., 2015; Kowalski et al., 2014; Zych et al., 2019b).

Recent research further reinforces the protective role of peer support, demonstrating its effectiveness in reducing the likelihood of involvement in both cyberbullying and cybervictimization (A. C. Baldry et al., 2015; Kowalski et al., 2019; Arató et al., 2022; Martín-Criado et al., 2021; Piazuelo-Rodríguez et al., 2024). Notably, a recent study by Malinowska-Cieślik et al. (2022) examined peer support across different cyberbullying roles, including cyberbully/victims, and found that peer support significantly reduced the likelihood of involvement exclusively for cybervictims. Moreover, Piazuelo-Rodríguez et al. (2024) show that this protective effect varies by gender: peer support is particularly salient for girls, for whom higher levels of perceived support substantially reduce the likelihood of cybervictimization, whereas the association is weaker among boys, suggesting a less pronounced protective influence in male adolescents.

### 1.3. The Present Study

Overall, previous research indicates that involvement in cyberbullying is influenced by multiple interacting individual, family, and school factors. Empathy, moral disengagement, antisocial behaviours, parental online monitoring, parental support, aggression toward teachers, and peer support have all been identified as significant predictors of cyberbullying involvement. However, the existing evidence remains fragmented, as most studies have examined these factors separately or have focused primarily on cyberbullies and cybervictims, providing a less comprehensive understanding of the phenomenon across roles. Despite this growing body of research, two important gaps remain.

First, adolescents involved as cyberbully/victims have received far less attention than cyberbullies and cybervictims across most ontogenetic, family, and school risk factors.

At the ontogenetic level, their empathic profiles remain largely unexplored (Zych et al., 2019a), evidence regarding moral disengagement is still limited (Marín-López et al., 2020), and antisocial behavior has not been directly investigated (Bayraktar et al., 2015, examined aggression and self-control, but not antisocial behavior specifically).

At the family level, no studies have examined cyberbully/victims in relation to parental online monitoring, while evidence regarding parental support remains relatively limited.

At the school level, no studies have systematically investigated whether aggression toward teachers is associated with involvement across the full spectrum of cyberbullying roles, and evidence regarding peer support among cyberbully/victims is still scarce (Malinowska-Cieślik et al., 2022).

Second, although several studies have reported gender differences in individual, family, and school risk factors, findings remain fragmented concerning patterns of involvement in cyberbullying categories and in pathway of risk factors across gender.

To address these unresolved issues, the present study adopted a socio-ecological perspective (Bronfenbrenner, 1979) to examine how individual-, family-, and school-level factors are associated with adolescents’ involvement in the three cyberbullying roles (Cyberbullies, Cybervictims, and Cyberbully/Victims) compared with adolescents not involved in cyberbullying, separately for boys and girls.

Furthermore, considering the scarce attention devoted to the Cyberbully/Victim group, and that previous research has consistently identified these adolescents as the subgroup showing the greatest psychosocial maladjustment, to further clarify the distinctive profile of this subgroup, direct comparisons between Cyberbully/Victims, Cyberbullies, and Cybervictims were also conducted across the examined psychosocial factors.

Based on previous literature, we expected that different pathways of involvement in cyberbullying for boys and girls. In particular, we hypothesized that high levels of affective empathy will be associated with girls’ involvement as cybervictims and/or cyberbully/victims. Conversely, we expected that boys involved as cyberbully/victims were more willing to report high levels of moral disengagement and deviant and aggressive behaviours towards teachers.

Given the inconsistent evidence regarding gender-related patterns reported in previous studies (A. C. Baldry et al., 2017; Quintana-Orts et al., 2024), we also examined whether the associations between psychosocial risk factors and cyberbullying involvement varied by gender. To this end, gender-stratified analyses were complemented by pooled multinomial logistic regression models that included gender × predictor interaction terms, enabling a formal evaluation of gender moderation.

## 2. Materials and Methods

### 2.1. Participants and Procedure

Participants were recruited from collaborating middle and high schools in the Campania and Calabria regions. Schools were invited to participate through existing collaborations and agreed voluntarily to take part in the study. All students with written parental informed consent were eligible to participate. The sample included 4543 adolescents (Mage = 13.97, SD = 2.08; 52.5% female).

The survey was administered during regular school hours under standardized conditions. Students were invited to participate and enrolled in the study after obtaining informed parental consent; participation was voluntary and anonymous. More specifically, participants were asked to complete an online self-assessment questionnaire containing questions about their use of new communication technologies over the previous six months. Before completing the questionnaire, participants were provided with a standardized definition of cyberbullying to ensure a common understanding of the study topic. The following definition was used: “Cyberbullying is an aggressive and intentional act, perpetrated by a group or individual, using electronic forms of contact, repeatedly over time against a victim who is unable to easily defend themselves” (Smith et al., 2008, p. 376).

Students completed the online checklist in computer labs in groups of 10–20, with assistance from trained researchers. Data were securely stored and processed in accordance with ethical guidelines for research involving minors. All study procedures were conducted in accordance with the guidelines of the Declaration of Helsinki and its subsequent amendments (2013). Furthermore, prior to data collection, approval from the Ethics Committee of the Department of Psychology (29/2015) was obtained.

### 2.2. Measures

The TABBY Improved online checklist (A. Baldry et al., 2018; Sorrentino et al., 2018) is a self-assessment tool developed to assess risk factors for youth involvement in cyberbullying and cybervictimization.

The instrument was developed following a review of the international literature (A. C. Baldry et al., 2015) and is conceptually informed by the socio-ecological framework (Bronfenbrenner, 1979) and the threat assessment approach (Borum et al., 1999). For the purposes of the present study, we selected the scales assessing ontogenetic-, family-, and school-level factors that were relevant to the study objectives. The measures included in the analyses are described below.

#### 2.2.1. Cyberbullying and Cybervictimization

The cyberbullying and cybervictimization scales were based on Willard’s (2007) taxonomy, which conceptualizes cyberbullying as five distinct online aggressive behaviours: flaming, denigration, impersonation, outing, and exclusion. Each behaviour was assessed using two parallel items, one referring to perpetration and the other to victimization.

Thus, the cyberbullying and cybervictimization scales assessed identical behavioural content, differing only in whether participants reported perpetrating and/or experiencing each behaviour.

Past six-month involvement in cyberbullying and cybervictimization was assessed using 10 items (five items each for perpetration, e.g., “I disclosed online private information or images without the person’s consent”, α = 0.86, and five items each for victimization, “I was actively engaged in excluding someone from an online group”α = 0.80). Participants rated each item on a 5-point frequency scale ranging from 1 (“never”) to 5 (“several times a week”). In order to create the Cyberbullying involvement categories variable, cyberbullying and cybervictimization scales were summed, and then, adopting a non-conservative criterion (Patchin & Hinduja, 2015; A. C. Baldry et al., 2017), categories were created as follows: students reporting never being involved either as cyberbullies and cybervictims were classified as not involved. Students who had never cyberbullied others, but had been cybevictimized at least once in the previous 6 months, were classified as cybervictims. Students who cyberbullied others at least once, but reported never being cybervictimized, were classified as cyberbullies. Students who were involved both as cyberbullies and cybervictims were classified as cyberbully/victims.

#### 2.2.2. Empathy

Empathic abilities were assessed using the Basic Empathy Scale (Jolliffe & Farrington, 2006; Albiero et al., 2009), a self-report instrument designed to assess adolescents’ cognitive and affective empathy. The scale comprises two subscales: Cognitive Empathy (9 items) assessing the ability to understand other people emotions (e.g., “I can understand my friend’s happiness when he/she does well at something”) and Affective Empathy (11 items) assessing the tendency to share others’ emotional experiences (e.g., “After being with a friend who is sad about something, I usually feel sad”). Items within each subscale were summed to obtain continuous scores, which were entered into the multinomial logistic regression analyses as continuous predictors. To facilitate the interpretation of the regression coefficients, all items were reverse-coded so that higher scores reflected lower cognitive and affective empathy. Internal consistency was satisfactory for both the Cognitive Empathy (α = 0.77) and Affective Empathy (α = 0.76) subscales.

#### 2.2.3. Moral Disengagement

Moral disengagement was assessed using the Moral Disengagement Scale (Bandura et al., 1996; Caprara et al., 2006), a self-report instrument designed to assess the cognitive mechanisms through which individuals justify or rationalize unethical behaviour. The scale consists of 32 items (e.g., “It is alright to fight to protect your friends”), rated on a 5-point Likert scale ranging from 1 = “Strongly disagree” to 5 = “Strongly agree”. Item scores were summed to obtain a continuous moral disengagement score, which was entered into the multinomial logistic regression analyses as a continuous predictor. Higher scores indicated higher levels of moral disengagement. Internal consistency was excellent (α = 0.94).

#### 2.2.4. Antisocial Behavior

Involvement in deviant behaviors was assessed using a five-item self-report scale measuring adolescents’ involvement in common deviant behaviours (e.g., *“I have damaged or stolen others’ property”*). Each item was scored dichotomously (0 = *No*, 1 = *Yes*). Item scores were summed to obtain a continuous score ranging from 0 to 5, with higher scores indicating greater involvement in antisocial behaviour. The resulting score was entered into the multinomial logistic regression analyses as a continuous predictor. Internal consistency was acceptable (α = 0.65).

#### 2.2.5. Aggression Toward Teachers

Students’ aggressive behaviors against teachers were measured using a four-item self-report scale measuring adolescents’ aggressive behaviours directed at teachers (e.g., “Have you teased your teachers online?”). Each item was scored dichotomously (0 = No, 1 = Yes). Item scores were summed to obtain a continuous score ranging from 0 to 4, with higher scores indicating greater aggression toward teachers. The resulting score was entered into the multinomial logistic regression analyses as a continuous predictor. Internal consistency was acceptable (α = 0.63).

#### 2.2.6. Perceived Social Support

Perceived social support was assessed using the Multidimensional Scale of Perceived Social Support (MSPSS; Zimet et al., 1988, 1990). The scale comprises three four-item subscales measuring perceived parental support (e.g., “I can talk about my problems with my family”), peer support (e.g., “I can count on my friends when things go wrong”), and significant other support (e.g., “There is a special person who is around when I am in need”). Participants responded on a seven-point Likert scale ranging from 1 (Strongly agree) to 7 (Strongly disagree). Items belonging to each subscale were summed to obtain continuous scores, which were entered into the multinomial logistic regression analyses as continuous predictors. Higher scores reflected lower levels of perceived parental (α = 0.91), peer (α = 0.92), and significant other support (α = 0.89). Accordingly, these variables are labelled in the regression tables as Low perceived parental support, Low perceived peer support, and Low perceived significant other support to facilitate interpretation.

#### 2.2.7. Parental Online Monitoring

Parental online monitoring was assessed using a three-item measure evaluating adolescents’ perceptions of their parents’ online supervision practices. The items assessed whether parents discussed Internet safety, established clear rules regarding Internet use, and monitored their children’s online activities. Participants responded on a five-point Likert scale ranging from 1 (*Always*) to 5 (*Never*). Item scores were summed to obtain a continuous score, which was entered into the multinomial logistic regression analyses as a continuous predictor. Higher scores reflected lower levels of parental online monitoring. Internal consistency was acceptable (α = 0.75).

### 2.3. Data Analyses

The data were analysed using IBM SPSS Statistics (Version 21.0; IBM Corp., Armonk, NY, USA). Multinomial regression analyses were performed separately for boys and girls to examine the associations between individual, parental and school-level risk factors in cyberbullying categories of involvement. Participants not involved in cyberbullying served as the reference category. Statistical significance was set at *p* < 0.05. Prior to conducting the multinomial logistic regression analyses, multicollinearity among the predictors was assessed using tolerance and variance inflation factor (VIF) statistics obtained from an auxiliary linear regression including the same predictors entered in the multinomial models. All tolerance values exceeded 0.50 and all VIF values were below 2, indicating that multicollinearity was not a concern. The assumption of linearity in the logit for the continuous predictors was assessed using the Box–Tidwell procedure. Although minor departures from strict linearity were observed for some predictors, these were small in magnitude and the original model specification was retained.

## 3. Results

### 3.1. Prevalence and Gender Differences in Cyberbullying Categories

Participants were classified into four mutually exclusive categories based on their involvement in cyberbullying over the past six months: Not Involved, Cyberbullies, Cyberbully/victims, and Cybervictims. Of the total sample, 60.1% (n = 2729) of participants were not involved in cyberbullying at all, 7.2% (N = 328) reported being a cyberbully, 19.4% (n = 882) were cybervictims and 13.3% (N = 604) were cyberbully/victims.

Among boys, 60.2% were not involved in cyberbullying at all, 14.1% were cybervictims, 15.3% were cyberbully/victims, and 10.4% were cyberbullies. Among female participants, 60.0% were not involved in cyberbullying, 4.4% reported being involved as cyberbullies, 24.2% reported being cybervictimized, and 11.5% were cyberbully/victims.

Gender differences in cyberbullying categories of involvement were investigated (see Table 1). 68.3% and 54.8% of all boys reported being only cyberbullies or cyberbully/victims respectively. Odds ratios indicate the existence of significant gender differences; girls were more likely to be cybervictims, while boys were more likely to be involved as cyberbullies and cyberbully/victims.

### 3.2. Direct Comparisons Between Cyberbullying Roles and Ontogenetic, Parental and School Risk Factors

In order to investigate the distinctive profile of this subgroup, direct comparisons between Cyberbully/Victims, Cyberbullies, and Cybervictims were conducted across the examined psychosocial factors (Table 2).

Cyberbullies (M = 20.07, SD = 7.09) reported low levels of affective empathy compared to both cybervictims (M = 14.97, SD = 6.98; F(1,1208) = 126.334, *p* < 0.001) and cyberbully/victims (M = 17.19, SD = 6.73; F(1,930) = 37.365). Cybervictims (M = 9.51, SD = 4.98) scored higher in cognitive empathy compared to cyberbullies (M = 10.95, SD = 5.16; F(1,1208) = 19.579, *p* < 0.001), while no significant differences in cognitive empathy were found between cyberbully/victims (M= 11.41, SD = 5.58) and cyberbullies (M = 10.95, SD = 5.16, F(1,930) = 1.519, *p* = n.s.).

Concerning moral disengagement, both cyberbullies (M = 83.94, SD = 24.45) and cyberbully/victims (M = 83.62, SD = 25.19) scored higher compared to cybervictims (M = 68.47, SD = 20.48), while no significant differences were found between cyberbullies (M = 83.94, SD = 24.45) and cyberbully/victims (M = 83.62, SD = 25.19; F(1,930) = 0.034, *p* = n.s.). The same pattern was also found concerning involvement in antisocial behaviours, highlighting no significant differences between cyberbullies (M = 0.98, SD = 1.17) and cyberbully/victims (M = 1.01, SD = 1.17; F(1,930) = 0.165, *p* = n.s.).

About parental-level risk factors, cyberbullies/victims (M = 10.59, SD = 6.42) report lower levels of perceived parental support than cyberbullies (M = 9.10, SD = 5.76) and cybervictims (M = 8.66, SD = 5.53). Concerning parental online monitoring a similar pattern, emerge, with cybervictims (M = 5.92, SD = 2.97) reporting high levels of parental online monitoring compared to cyberbullies (M = 7.50, SD = 2.76; F(1,1208) = 69.618, *p* < 0.001) and cyberbully/victim (M = 7.20, SD = 2.81; F(1,1484) = 68.522, *p* < 0.001). No differences were found between cyberbullies and cyberbully/victims (F(1,930) = 2.478, *p* = n.s.).

Regarding peer and school level risk factors, cyberbully/victims (M = 0.84, SD = 1.09) reported higher levels of aggression towards teachers than cyberbullies (M = 0.63, SD = 0.92; F(1,930) = 8.885, *p* < 0.01). With regard to perceived peer support, cyberbully/victims (M = 11.12, SD = 6.13) significant perceive low levels of peer support than cyberbullies (M = 9.57, SD = 5.89; F(1,930) = 13.998, *p* < 0.001). The same pattern was found also concerning levels of perceived social support from Significant Other.

### 3.3. Multinomial Logistic Regression

#### 3.3.1. Risk Factors for Involvement in Cyberbullying Categories Among Boys

Cybervictims vs. not involved

Significant predictors for cybervictimization, at the individual level, were high levels of affective (B = −0.027, OR = 0.97, *p* = 0.017) and cognitive empathy (B = −0.030, OR = 0.97, *p* = 0.04) (Table 3).

Concerning family-level risk factors, cybervictims were more likely to report low levels of perceived parental support (B = 0.049, OR = 1.050, *p* = 0.005) and high levels of online monitoring (B = −0.056, OR = 0.945, *p* = 0.011) compared to male participants not involved in cyberbullying.

At the school level, cybervictimization was predicted by aggression toward teachers (B = 0.281, OR = 1.32, *p* = 0.009), low levels of perceived peer support (β = 0.078, OR = 1.081, *p* = 0.000), and high levels of perceived other significant support (B = −0.056, OR = 0.945, *p* = 0.003).

Cyberbully/victims vs. not involved

Cyberbully/victims reported high levels of affective empathy (B = −0.040, OR = 0.961, *p* = 0.001), moral disengagement (B = 0.017, OR = 1.017, *p* = 0.000), and antisocial behaviors (B = 0.521, OR = 1.683, *p* = 0.000). Concerning the family level, they reported low levels of perceived parental support (B = 0.035, OR = 1.035, *p* = 0.041), while at the school level, compared to students not involved, cyberbully/victims scored higher in aggression towards teachers (B = 0.517, OR = 1.676, *p* = 0.000).

Cyberbully vs. not involved

Involvement in cyberbullying was significantly predicted, at the individual level, by low levels of affective empathy (B = 0.036, OR = 1.036, *p* = 0.005), high levels of cognitive empathy (B = −0.040, OR = 0.961, *p* = 0.014), moral disengagement (B = 0.018, OR = 1.019, *p* = 0.000), and antisocial behaviors (B = 0.419, OR = 1.521, *p* = 0.000) compared to not involved. With regard to family level risk factors, cyberbullies reported lower levels of perceived parental support (B = 0.043, OR = 1.044, *p* = 0.028) and online monitoring (B = 0.058, OR = 1.060, *p* = 0.037) compared to male participants not involved in cyberbullying. At the school level, involvement as a cyberbully was predicted by aggression toward teachers (B = 0.319, OR = 1.376, *p* = 0.002) and high levels of perceived social support from a significant other (B = −0.046, OR = 0.955, *p* = 0.035).

#### 3.3.2. Risk and Protective Factors of Group Membership Among Girls

Cybervictims vs. not involved

Compared to not involved, girls involved as cybervictims reported high levels of affective empathy (B = −0.030, OR = 0.971, *p* = 0.001) and moral disengagement (B = 0.011, OR = 1.011, *p* = 0.000) (Table 3). At the family level, compared to not involved, cybervictims scored lower in perceived parental social support (B = 0.053, OR = 1.054, *p* = 0.000) and higher in parental online monitoring (B = −0.050, OR = 0.952, *p* = 0.005). At the school levels, significant predictors of cybervictimization are aggressions towards teachers (B = 0.326, OR = 1.386, *p* = 0.002), low levels of perceived social support from peers (B = 0.053, OR = 1.054, *p* = 0.000), and high levels of perceived social support from a significant other (B = −0.050, OR = 0.952, *p* = 0.002).

Cyberbully/victims vs. not involved

Cyberbully/victims, at the individual level, were more likely to report high levels of affective empathy (B = −0.041, OR = 0.960, *p* = 0.002), moral disengagement (B = 0.023, OR = 1.023, *p* = 0.000), and involvement in antisocial behaviors (B = 0.423, OR = 1.527, *p* = 0.000) compared to their female counterparts not involved.

At the family level, only low levels of perceived parental support were significantly associated with cyberbully/victims involvement (B = 0.099, OR = 1.104, *p* = 0.000). Whereas, at the school level, cyberbully/victims were more likely to be involved in aggression toward teachers (B = 0.840, OR = 2.317, *p* = 0.000), and to report low levels of perceived social support from peers (B = 0.053, OR= 1.055, *p* = 0.000), and high levels of perceived social support from a significant other (B = −0.097, OR = 0.907, *p* = 0.000) than not involved.

Cyberbullies vs. not involved

Cyberbullies, at the individual level, were more likely to report low levels of affective empathy (B = 0.053, OR = 1.055, *p* = 0.002), high moral disengagement (B = 0.021, OR = 1.021, *p* = 0.000), and involvement in antisocial behaviors (B = 0.605, OR = 1.830, *p* = 0.000) compared to their female counterpart not involved. None of the family-level risk factors was significantly associated with cyberbullying involvement. Concerning the school level risk factors, girls engaged in cyberbullying scored higher in aggression toward teachers compared to those not involved (B = 0.774, OR = 2.169, *p* = 0.000).

#### 3.3.3. Gender Moderation in Cyberbullying Categories of Involvement

To further explore whether the associations between the examined predictors and cyberbullying involvement varied according to gender, an additional multinomial logistic regression model including interaction terms between sex and each continuous predictor was estimated (Table 4). No significant interaction effects emerged for the cybervictim group, indicating comparable associations across boys and girls. Among cyberbully/victims, significant interactions were observed for parental support (B = 0.064, OR = 1.07, *p* = 0.006), aggression toward teachers (B = 0.324, OR = 1.382, *p* = 0.022), and significant other support (B = −0.096, OR = 0.91, *p* = 0.001). Specifically, the associations between lower parental support and aggression toward teachers and cyberbully/victim involvement were stronger among girls than among boys, whereas the association with lower significant-other support was stronger among boys. Among cyberbullies, only the interaction between sex and aggression toward teachers reached statistical significance, indicating a stronger association among girls (B = 0.455, OR = 1.58, *p* = 0.01). The remaining interaction terms were not statistically significant, suggesting that the associations between empathy, moral disengagement, antisocial behaviour, peer support, and parental online monitoring and cyberbullying involvement were largely comparable across genders.

## 4. Discussion

Understanding the dynamics of adolescent involvement in cyberbullying requires an integrative socio-ecological perspective, as these behaviors emerge from the interplay of individual dispositions, family processes, and school-related experiences (Bronfenbrenner, 1979; Quintana-Orts et al., 2025). Previous research has often ignored the subgroup of cyberbully/victims, even though evidence has highlighted that they are at greater risk of developing psychosocial difficulties and engaging in deviant behaviors (Tintori et al., 2025; Quintana-Orts et al., 2024; Kennedy, 2021; A. C. Baldry et al., 2019a). The present study aims to extend this literature by examining the combined role of individual (i.e., empathy, moral disengagement, antisocial behavior), family (i.e., parental monitoring and perceived support), and school dimensions (i.e., aggression towards teachers, perceived peer and significant other support) in predicting involvement in distinct cyberbullying roles, by accounting for the existence of a different pattern of risk factors across gender. This multilevel approach offers a detailed understanding of how risk and protective factors influence trajectories of involvement in cyberbullying, cybervictimization and cyberbullying/victimization focusing on gender specific trajectories.

Analyses aimed at comparing cyberbullying involvement categories showed that cyberbullies and cyberbully/victims did not differ in moral disengagement, antisocial behavior, and cognitive empathy, indicating a shared pattern of ontogenetic risk factors, highlighting reduced sensitivity to guilt and a propensity to act antisocially, consistent with moral disengagement operating as a shared cognitive mechanism that fuels aggression (Pornari & Wood, 2010).

Distinctive features of the cyberbully/victims group lie in their greater involvement in aggression towards teachers than cyberbullies, lower levels of affective empathy than cybervictims, and low levels of perceived parents, peers and significant others support than cyberbullies. These results suggest that involvement as cyberbully/victims may reflect the accumulation of vulnerabilities across multiple developmental contexts rather than the presence of a single dominant risk factor. This interpretation is further supported by evidence on polyvictimization and polyaggression, indicating that experiences of victimization and aggression can be mutually reinforcing and generalize across different interpersonal contexts (Finkelhor et al., 2007; Mitchell et al., 2018; Semenza, 2021; Varela Torres et al., 2021).

Concerning our second aim, that is analyzing pathways of involvement in cyberbullying categories separately for boys and girls, our results underlined a different pattern across boys and girls who were involved as cybervictims. At the individual level, males involved as cybervictims are more likely to report low levels of both cognitive and affective empathy. Moving to girls, they showed higher levels of affective empathy and moral disengagement compared to those not involved.

Concerning the family-level risk factors, our results showed that both male and female cybervictims perceive lower levels of parental social support and higher levels of parental online monitoring than students not involved in cyberbullying.

At the school-level risk factors, both male and female cybervictims reported involvement in aggression towards schoolteachers, as well as low levels of perceived social support from both peers and significant other.

Interestingly, patterns of individual and family-level risk factors for involvement as cyberbully/victims were similar across gender. Among both boys and girls, cyberbully/victims report high levels of affective empathy, moral disengagement, antisocial behaviors, and low levels of perceived parental support. A different pattern emerged at the school level regarding perceived social support, with female cyberbully/victims reporting low levels of peer support but high levels of perceived social support from significant other.

Examining gender paths in cyberbullying involvement, several interesting findings emerged. At the individual level, both girls and boys reported low levels of affective empathy, high moral disengagement, and involvement in deviant behaviors. However, only male cyberbullies exhibited higher levels of cognitive empathy than students not involved. Among both males and females, cyberbullies were more likely to be involved in aggressive behaviors against teachers. Still, only male cyberbullies reported high levels of perceived social support from a significant other. In contrast, male participants not involved in cyberbullying at all did not report any perceived social support. Finally, the results about the family-level risk factors showed the strongest gendered pathway in cyberbullying involvement, with male cyberbullies reporting low levels of perceived social support from parents and low levels of parental online monitoring.

Our findings reveal distinct empathic profiles across different roles in cyberbullying categories of involvement for boys and girls. In particular, only male cyberbullies scored higher in cognitive empathy. This pattern partially aligns with the international literature (e.g., Zych et al., 2019b). In this regard, evidence suggested that young men are more likely to engage in cognitive-mentalizing strategies, whereas young women tend to exhibit greater emotional resonance (Christov-Moore et al., 2014; Ang & Goh, 2010), thus supporting our result concerning high levels of cognitive empathy among boys cyberbullies. This finding aligns with De Waal’s (2008) assertion that cognitive perspective-taking, when not accompanied by affective engagement, can lead to both prosocial and antisocial outcomes.

Additionally, girls involved as cybervictims or cyberbully/victims showed higher levels of affective empathy than those not involved in cyberbullying. This pattern aligns with existing literature showing that girls typically score higher on measures of emotional resonance and affective mirroring (Davis, 1983; Hoffman, 2000). It also supports previous studies examining the connections between affective empathy and cybervictimization (Fabris et al., 2022; Zych et al., 2019b). At first glance, the relationship between being involved as cyberbully/victims and exhibiting high levels of affective empathy may seem counterintuitive. However, recent research indicates that affective empathy consists of two sub-dimensions: affective resonance and affective dissonance (Vachon et al., 2014; Vachon & Lynam, 2016). Affective dissonance, which is associated with aggressive and externalizing behaviors, may explain situations in which high levels of affective empathy lead to aggression. Although the present study did not directly assess passive-aggressive interpersonal style or relational aggression, the pattern observed among female cyberbully/victims is also consistent with this interpretation. Specifically, girls in the dual role combined high affective empathy with elevated moral disengagement, antisocial behaviour, and aggression toward teachers. Rather than reflecting a deficit in emotional sensitivity, this profile may indicate an increased ability to perceive others’ emotional states while simultaneously relying on cognitive mechanisms that justify or neutralize aggressive behaviour. Within online environments, where indirect, relational, and less confrontational forms of aggression are facilitated by anonymity and reduced social accountability, these characteristics may favour passive-aggressive strategies rather than direct physical confrontation. Future studies should therefore examine whether passive-aggressive interpersonal styles and relational aggression represent additional mechanisms underlying female involvement in the cyberbully/victim role.

Our findings also showed an interesting pattern concerning cyberbullying categories of involvement and moral disengagement. In particular, both cyberbullies and cyberbully/victims are more likely to report high levels of moral disengagement compared to students not involved. This trend, consistent with previous studies (Lo Cricchio et al., 2021; Killer et al., 2019; Liu et al., 2023), was found among both male and female participants. A specific gender pattern concerned girls involved as cybervictims, who were found to show higher levels of moral disengagement compared to girls participants not involved at all in cyberbullying. This finding is consistent with previous research suggesting a possible gender-specific pathway for girls, according to which moral disengagement may serve as a psychological defense mechanism, enabling victims to cope with the emotional consequences of victimization by justifying their experiences (Allison & Bussey, 2017; Luo & Bussey, 2019).

In line with previous studies (Holfeld & Leadbeater, 2015; Kim et al., 2017; Sticca et al., 2013; You & Lim, 2016), both cyberbullies and cyberbully/victims were more willing to report greater involvement in antisocial behaviors than students not involved. This pattern is particularly notable among those in the dual role and is consistent across genders. These results suggest that victimization and perpetration dynamics may alternate and spill over across social contexts (Varela Torres et al., 2021), consistent with theoretical models of poly-victimization and poly-aggression (Semenza, 2021; Mitchell et al., 2018).

Our results underlined the crucial role of aggression toward teachers as a common risk factor for all cyberbullying categories of involvement across participants ‘gender. Although very few studies investigated the association between cyberbullying and aggression toward schoolteachers, we should speculate that, according to the poly-aggression theory (Mitchell et al., 2018; Semenza, 2021; Finkelhor et al., 2007), both cyberbullies and cyberbully/victims could transfer the aggressive pattern learned in peer relationships also in conflictual and violent behaviors against their teachers. While acknowledging the cross-sectional nature of our study, one possible interpretation is that cybervictims may exhibit aggressive behaviors toward their teachers as a consequence of perceiving them as unable to effectively recognize and manage cybervictimization incidents (Sorrentino et al., 2026).

Our study’s findings indicate that types of perceived social support showed a differential pattern across various forms of involvement in cyberbullying among boys and girls.

Concerning cybervictimization, our results showed that both boys and girls reported lower levels of perceived peer and parental support, confirming previous evidence that low levels of such types of perceived support constitute a risk factor for online victimization (Fanti et al., 2012; Kowalski et al., 2014; N. Marengo et al., 2021). These relationships were also found among girls involved as cyberbully/victims, corroborating previous studies underlying that cyberbully/victims were characterized by family conflicts, weaker parental bonds, and lower family cohesion (Bayraktar et al., 2015; Kokkinos et al., 2016; Buelga et al., 2017). Interestingly, on the other hand, girls involved both as cybervictims and cyberbully/victims scored higher in perceived social support from significant others. In this regard, we could speculate that both female cybervictims and cyberbully/victims could benefit from the presence of a defender, who may provide support, ask for help from peers, teachers, or other adults, or try to stop the aggression directly (Sarmiento et al., 2019). Similarly, the high levels of perceived social support from a significant other reported by boys involved as cyberbullies could reflect the group dynamics within the cyberbullying phenomenon. This suggests that cyberbullies might receive positive feedback from their peers, acting as a form of reinforcement (Sarmiento et al., 2019).

Concerning parental online monitoring, a common pattern among male and female cybervictims emerged, with higher levels of parental online monitoring than participants not involved. Whereas only male cyberbullies exhibited low levels of parental monitoring.

Such results concerning cyberbullies seem to support that a lack of parental online monitoring represents a significant risk factor for youth involvement in cyberbullying (Gómez et al., 2017; Hood & Duffy, 2018; Vazsonyi et al., 2017), while, on the contrary, a high level of parental monitoring was found to be associated with cybervictimization across gender. The latter finding aligns with previous research suggesting that insufficient parental online monitoring constitutes a significant risk factor for youth engagement in cyberbullying (Gómez et al., 2017; Hood & Duffy, 2018). Conversely, the association between high parental monitoring and cybervictimization may indicate a paradoxical effect and could, in line with A. C. Baldry et al. (2019a), reflect that sons’ cybervictimization leads to increased parental concern and online supervision.

An additional contribution of the present study concerns the formal examination of gender moderation. Most associations between psychosocial predictors and cyberbullying involvement were comparable across boys and girls.

Gender moderated levels of perceived parental and significant other social support among girls involved as cyberbully/victims, indicating low levels of perceived social support. The observed gender difference in parental support is consistent with recent evidence highlighting the greater relevance of family processes for girls’ involvement in cyberbullying (Pan et al., 2025).

Aggression toward teachers was significantly moderated by gender among cyberbullies and cyberbully/victims, with girls showing greater involvement compared with males. To our knowledge, this pattern has not been previously reported in the cyberbullying literature; however, our results seem to underline the importance of further investigating how involvement in aggression towards teachers could affect cyberbullying involvement, also taking into account gender differences.

### Limitations and Future Directions

Despite the strengths of this study—such as the large sample size, the integration of multiple ecological levels, and the focus on the underexplored cyberbully/victims group—several limitations should be acknowledged. First, the cross-sectional design does not allow for causal inferences. Although the multinomial analysis identifies patterns of association between risk factors and cyberbullying roles, it is not possible to determine the directionality of these relationships. For instance, higher parental monitoring may act as a protective factor, but it may also increase in response to adolescents’ exposure to cybervictimization. Longitudinal studies are needed to examine reciprocal processes and developmental trajectories over time. Second, all variables were assessed via adolescent self-report, which may introduce common-method bias, social desirability effects, and inaccuracies in recall of online behaviors. Future research would benefit from a multi-informant approach that incorporates reports from parents, teachers, and peers, as well as behavioral or digital-trace data where appropriate. Third, although the socio-ecological framework guided the selection of individual, family, and school variables, some relevant ecological dimensions were not included. In particular, factors from the meso- and exosystem—such as the quality of school–family communication, broader community involvement, neighborhood characteristics, and school climate—could further clarify the contextual influences on cyberbullying roles. Likewise, specific online contextual variables (e.g., patterns of social media use, exposure to online risks, digital literacy, and platform-specific affordances) were not examined, despite their growing relevance in adolescents’ daily lives. Another limitation could regard our participants shared contextual environment which should result in potentially correlated observations and underestimated standard errors. Future studies should consider the clustering effect when participants are recruited.

Finally, the antisocial behaviour and aggression toward teachers’ scales showed only moderate internal consistency. However, both measures consisted of a small number of dichotomous items, a condition known to attenuate Cronbach’s alpha (Gliem & Gliem, 2003). Consequently, the observed associations should be interpreted with some caution, as measurement error may have led to conservative estimates of the relationships examined. In addition, although the assumption of linearity in the logit was formally evaluated, some continuous predictors showed minor departures from strict linearity. While these deviations were not considered substantial enough to warrant re-specification of the multinomial regression models, future research could examine potential non-linear associations using more flexible modelling approaches.

Our findings enhance the existing understanding of gender-specific patterns in the various categories of cyberbullying involvement. They provide valuable insights for developing more targeted and effective prevention strategies to reduce youth violence globally (Semenza, 2021). Rather than focusing solely on the binary roles of aggressor and victim, this approach considers contemporary involvement in multiple forms of aggression and victimization across different relational contexts (Finkelhor et al., 2007; Hamby et al., 2018). In this regard, prevention programs could be developed to present multiple modules and activities to fully target all youth involved in cyberbullying. Some of these modules could be cross-cutting and affect both genders and focus on the prevention and the overlap of aggressive and violent behaviors in different social contexts, such as violence toward teachers and deviant behaviors, thus promoting consistent norms against aggression both online and offline. Particular attention should be paid to maladaptive cognitive mechanisms and “empathic dysregulation” underlying aggressive behavior. The elevated levels of moral disengagement observed in this study suggest the presence of cognitive scripts that justify violence. In such cases, adolescents may internalize narratives and neutralization strategies—such as minimizing harm, dehumanizing the target, or attributing blame to the victim—similar to those documented in traditional bullying. These mechanisms serve not only to legitimize aggressive conduct but also to distort the interpretation of one’s own victimization experiences (Thornberg & Jungert, 2014; Thornberg et al., 2018; Schacter et al., 2015). Consequently, preventive interventions should include components explicitly aimed at identifying, deconstructing, and restructuring such cognitive scripts, promoting alternative ways of understanding conflict and personal responsibility. Alongside this, the findings on empathy indicate gender- and role-specific pathways, suggesting that the capacity to adopt others’ cognitive perspectives—when not supported by emotional resonance—may be deployed for both prosocial and antisocial purposes (De Waal, 2008; Perry et al., 2017). Interventions designed to integrate emotional resonance with cognitive understanding may, particularly for boys, help reduce the strategic and manipulative use of empathy, fostering, instead, a more adaptive regulation of emotions and social interactions. Regarding girls, especially when involved as cyberbully/victims or cyberbullies, interventions should be designed to detect and reduce their levels of dissonant affective empathy. Concerning our findings on perceived peer and parental social support several activities should be implemented, such as promoting peer educators training at school in order to create a sort of ‘buddy’ advice service to seek help and support when at risk of being involved in cyberbullying. To promote parental involvement and support, multi-level, multi-component prevention programs are necessary. In this regard, schools should act as facilitator by promoting and organizing meetings with experts to raise awareness on cyberbullying roles and features and to engage parents in supporting, monitoring and educating their sons to a safe Internet use. Teachers and school involvement in the promotion of such activities could also foster the quality of the students-teachers relationship, maybe contributing also to an increased level of perceived social support from significant others by students involved in cyberbullying.

## 5. Conclusions

Our findings highlight the importance of continuing to investigate the psychosocial factors associated with cyberbullying involvement using a comprehensive socio-ecological framework. In particular, the present study provides further evidence that adolescents involved as Cyberbully/Victims represent a particularly vulnerable subgroup characterized by a more maladaptive psychosocial profile than adolescents occupying a single role. By analisying risk factors for the involvement in cyberbullying categories separately for boys and girls, the study offers a more nuanced characterization of the dual-role profile and identifies specific individual-, family-, and school-level factors associated with this subgroup. Furthermore, the additional interaction analyses indicate that most psychosocial risk factors operate similarly across boys and girls. These findings suggest that prevention and intervention programmes should primarily target the common psychosocial mechanisms underlying cyberbullying involvement, while also considering gender-specific pathways. Overall, adopting a multi-level socio-ecological approach remains essential for identifying adolescents at greater risk—particularly Cyberbully/Victims—and for developing prevention strategies that simultaneously address individual, family, peer, and school contexts.

## Figures and Tables

**Table 1:** Gender differences across cyberbullying involvement categories.

		Males	Females
Not involved	N	1300	1429
(vs. other)	%	47.6	52.4
	OR (95%CI)	0.99 (0.88–1.12)	
Cybervictims	N	305	577
(vs. other)	%	34.6	65.4
	OR (95%CI)	1.94 *** (1.67–2.27)	
Cyberbully/victims	N	331	273
(vs. other)	%	54.8	45.2
	OR (95%CI)	0.72 *** (0.60–0.85)	
Cyberbullies	N	224	104
(vs. other)	%	68.3	31.7
	OR (95%CI)	0.39 *** (0.31–0.50)	

Notes. Each category of involvement in cyberbullying was compared against all the rest of the sample. *** *p* < 0.001.

**Table 2:** Direct comparisons between cyberbullying roles and ontogenetic, parental and school risk factors.

		M (S.D.)	F_(1,1208)_		M (S.D.)	F_(1,1484)_		M (S.D.)	F_(1,930)_
Low affective empathy	CV	14.97 (6.98)	126.334 ***	CB/CV	17.19 (6.73)	37.319 ***	CB	20.07 (7.09)	37.365 ***
	CB	20.07 (7.09)		CV	14.97 (5.58)		CB/CV	17.19 (6.73)	
Low cognitive empathy	CV	9.51 (4.98)	19.579 ***	CB/CV	11.41 (5.58)	47.248 ***	CB	10.95 (5.16)	1.519
	CB	10.95 (5.16)		CV	9.51 (4.98)		CB/CV	11.41 (5.58)	
Moral disengagement	CV	68.47 (20.48)	122.325 ***	CB/CV	83.62 (25.19)	162.424 ***	CB	83.94 (24.45)	0.034
	CB	83.94 (24.45)		CV	68.47 (20.48)		CB/CV	83.62 (25.19)	
Antisocial behaviors	CV	0.31 (0.68)	145.879 ***	CB/CV	1.01 (1.17)	205.473 ***	CB	0.98 (1.17)	0.165
	CB	0.98 (1.17)		CV	0.32 (0.68)		CB/CV	1.01 (1.17)	
Low perceived parental support	CV	8.66 (5.53)	1.476	CB/CV	10.59 (6.42)	38.224 ***	CB	9.10 (5.76)	12.287 ***
	CB	9.10 (5.76)		CV	8.66 (5.53)		CB/CV	10.59 (6.42)	
Low online parental monitoring	CV	5.92 (2.97)	69.618 ***	CB/CV	7.20 (2.81)	68.522 ***	CB	7.50 (2.76)	2.478
	CB	7.50 (2.76)		CV	5.92 (2.97)		CB/CV	7.20 (2.81)	
Aggression toward teachers	CV	0.27 (0.61)	63.952 ***	CB/CV	0.84 (1.09)	168.609 ***	CB	0.63 (0.92)	8.885 **
	CB	0.63 (0.92)		CV	0.27 (0.61)		CB/CV	0.84 (1.09)	
Low perceived peer support	CV	10.27 (5.82)	3.502	CB/CV	11.12 (6.13)	7.254 **	CB	9.57 (5.89)	13.998 ***
	CB	9.57 (5.89)		CV	10.27 (5.82)		CB/CV	11.12 (6.13)	
Low perceived significant other support	CV	8.24 (5.21)	2.839	CB/CV	9.90 (6.39)	30.317 ***	CB	8.82 (5.55)	6.714 *
	CB	8.82 (5.55)		CV	8.24 (5.21)		CB/CV	9.90 (6.39)	

Note. * *p* < 0.05, ** *p* < 0.01, *** *p* < 0.001.

**Table 3:** Multinomial logistic regression analyses predicting involvement in cyberbullying categories (Cybervictims, Cyberbully/Victims, and Cyberbullies) among boys and girls compared with adolescents not involved in cyberbullying. Regression coefficients (B), standard errors (SE), odds ratios (OR), and 95% confidence intervals are reported. Significant predictors are indicated by asterisks.

			B (S.E.)	Exp (B)	95% Confidence Interval for OR			B (S.E.)	OR	95% Confidence Interval for OR	
					Lower Bound	Upper Bound				Lower Bound	Upper Bound
Boys							Girls				
	Cybervictims										
		Affective empathy	−0.03 (0.01)	0.97 *	0.95	0.99		−0.03 (0.01)	0.97 **	0.95	0.99
		Cognitive empathy	−0.03 (0.02)	0.97 *	0.94	0.99		−0.009 (0.01)	0.99	0.97	1.02
		Moral Disengagement	0.004 (0.003)	1.00	0.99	1.01		0.01 (0.003)	1.01 ***	1.01	1.02
		Antisocial behaviors	0.11 (0.09)	1.12	0.94	1.33		0.11 (0.10)	1.11	0.91	1.36
		Low perceived parental support	0.05 (0.02)	1.05 *	1.02	1.09		0.05 (0.01)	1.05 ***	1.03	1.08
		Low online parental monitoring	−0.06 (0.02)	0.95 *	0.91	0.99		−0.05 (0.02)	0.95 *	0.92	0.99
		Aggression towards teachers	0.28 (0.11)	1.32 **	1.07	1.64		0.33 (0.11)	1.39 **	1.13	1.71
		Low perceived peer support	0.08 (0.02)	1.08 ***	1.05	1.12		0.05 (0.01)	1.05 ***	1.03	1.08
		Low perceived significant other support	−0.06 (0.02)	0.95 **	0.91	0.98		−0.05 (0.02)	0.95 **	0.92	0.98
	Cyberbully/victims										
		Affective empathy	−0.04 (0.01)	0.96 **	0.94	0.99		−0.04 (0.01)	0.96 **	0.94	0.99
		Cognitive empathy	0.02 (0.02)	1.02	0.99	1.05		0.002 (0.02)	1.00	0.97	1.04
		Moral Disengagement	0.02 (0.003)	1.02 ***	1.01	1.02		0.02 (0.004)	1.02 ***	1.02	1.03
		Antisocial behaviors	0.52 (0.08)	1.68 ***	1.45	1.95		0.42 (0.11)	1.53 ***	1.24	1.88
		Low perceived parental support	0.04 (0.02)	1.04 *	1.00	1.07		0.09 (0.02)	1.10 ***	1.07	1.14
		Low online parental monitoring	0.03 (0.02)	1.03	0.98	1.08		0.01 (0.03)	1.01	0.96	1.06
		Aggression towards teachers	0.52 (0.09)	1.68 ***	1.41	2.00		0.84 (0.11)	2.32 ***	1.87	2.87
		Low perceived peer support	0.03 (0.02)	1.03	0.99	1.07		0.05 (0.02)	1.06 **	1.02	1.09
		Low perceived significant other support	−0.001 (0.02)	0.99	0.96	1.04		−0.09 (0.02)	0.91 ***	0.87	0.95
	Cyberbullies										
		Affective empathy	0.04 (0.01)	1.04 **	1.01	1.06		0.05 (0.02)	1.06 **	1.02	1.09
		Cognitive empathy	−0.04 (0.02)	0.96 *	0.93	0.99		−0.02 (0.02)	0.98	0.93	1.03
		Moral Disengagement	0.02 (0.003)	1.02 ***	1.01	1.03		0.02 (0.005)	1.02 ***	1.01	1.03
		Antisocial behaviors	0.42 (0.08)	1.52 ***	1.30	1.78		0.61 (0.13)	1.83 ***	1.41	2.38
		Low perceived parental support	0.04 (0.02)	1.04 *	1.01	1.08		0.04 (0.03)	1.04	0.99	1.09
		Low online parental monitoring	0.06 (0.03)	1.06 *	1.00	1.12		0.02 (0.04)	1.02	0.94	1.09
		Aggression towards teachers	0.32 (0.10)	1.38 **	1.12	1.69		0.77 (0.14)	2.17 ***	1.64	2.88
		Low perceived peer support	0.01 (0.02)	1.01	0.98	1.06		−0.02 (0.03)	0.98	0.93	1.04
		Low perceived significant other support	−0.05 (0.02)	0.96 *	0.916	0.997		−0.05 (0.03)	0.95	0.89	1.02
			*R*^2^ = 0.21 (Cox and Snell). 0.24 (Nagelkerke). *χ*^2^_(27)_ = 512.82 ***					*R*^2^ = 0.16 (Cox and Snell). 0.19 (Nagelkerke). *χ*^2^_(27)_ = 428.08 ***			

Note. * *p* < 0.05, ** *p* < 0.01, *** *p* < 0.001.

**Table 4:** Gender moderation analyses.

		B (S.E.)	OR	95% Confidence Interval for OR	
				Lower Bound	Upper Bound
Cybervictims					
	Gender	−0.10 (0.35)	0.90	0.45	1.79
	Affective Empathy	−0.03 (0.01)	0.97 *	0.95	0.99
	Cognitive Empathy	−0.03 (0.02)	0.97 *	0.94	0.99
	Moral Disengagement	0.004 (0.003)	1.00	0.99	1.01
	Antisocial Behaviors	0.11 (0.09)	1.12	0.93	1.33
	Low perceived parental support	0.05 (0.02)	1.05 **	1.02	1.09
	Low online parental monitoring	−0.06 (0.02)	0.95 *	0.91	0.99
	Aggression towards teachers	0.28 (0.11)	1.32 **	1.07	1.64
	Low perceived peer support	0.08 (0.02)	1.08 ***	1.05	1.12
	Low perceived significant other support	−0.06 (0.02)	0.95 **	0.91	0.98
	Sex x Affective Empathy	−0.002 (0.02)	0.99	0.97	1.03
	Sex x Cognitive Empathy	0.02 (0.02)	1.02	0.98	1.06
	Sex x Moral Disengagement	0.007 (0.004)	1.01	0.99	1.02
	Sex x Antisocial Behaviors	−0.004 (0.14)	0.99	0.76	1.30
	Sex x Low perceived parental support	0.003 (0.02)	1.00	0.96	1.05
	Sex x Low online parental monitoring	0.006 (0.03)	1.01	0.95	1.06
	Sex x Aggression towards teachers	0.05 (0.15)	1.05	0.78	1.41
	Sex x Low perceived peer support	−0.03 (0.02)	0.98	0.94	1.02
	Sex x Low perceived significant other support	0.006 (0.03)	1.01	0.96	1.06
Cyberbully/victims					
	Gender	−0.06 (0.45)	0.94	0.39	2.27
	Affective Empathy	−0.04 (0.01)	0.96 ***	0.94	0.99
	Cognitive Empathy	0.02 (0.02)	1.02	0.99	1.05
	Moral Disengagement	0.02 (0.003)	1.02 ***	1.01	1.02
	Antisocial Behaviors	0.52 (0.08)	1.68 ***	1.45	1.95
	Low perceived parental support	0.04 (0.02)	1.04 *	1.00	1.07
	Low online parental monitoring	0.03 (0.02)	1.03	0.98	1.08
	Aggression towards teachers	0.52 (0.09)	1.68 ***	1.41	2.00
	Low perceived peer support	0.03 (0.02)	1.03	0.99	1.07
	Low perceived significant other support	−0.001 (0.02)	0.99	0.96	1.04
	Sex x Affective Empathy	−0.001 (0.02)	0.99	0.96	1.04
	Sex x Cognitive Empathy	−0.02 (0.02)	0.98	0.94	1.03
	Sex x Moral Disengagement	0.006 (0.005)	1.01	0.99	1.02
	Sex x Antisocial Behaviors	−0.09 (0.13)	0.91	0.70	1.17
	Sex x Low perceived parental support	0.06 (0.02)	1.07 **	1.02	1.12
	Sex x Low online parental monitoring	−0.02 (0.04)	0.98	0.92	1.05
	Sex x Aggression towards teachers	0.32 (0.14)	1.38 *	1.05	1.82
	Sex x Low perceived peer support	0.02 (0.03)	1.02	0.97	1.08
	Sex x Low perceived significant other support	−0.09 (0.03)	0.91 ***	0.86	0.96
Cyberbullies					
	Gender	−0.66 (0.58)	0.52	0.17	1.63
	Affective Empathy	0.04 (0.01)	1.04	1.01	1.06
	Cognitive Empathy	−0.04 (0.02)	0.96	0.93	0.99
	Moral Disengagement	0.02 (0.003)	1.02	1.01	1.03
	Antisocial Behaviors	0.42 (0.08)	1.52	1.29	1.78
	Low perceived parental support	0.04 (0.02)	1.04	1.01	1.08
	Low online parental monitoring	0.06 (0.03)	1.06	1.00	1.12
	Aggression towards teachers	0.32 (0.10)	1.38	1.12	1.69
	Low perceived peer support	0.01 (0.02)	1.01	0.98	1.06
	Low perceived significant other support	−0.05 (0.02)	0.96	0.92	0.99
	Sex x Affective Empathy	0.02 (0.02)	1.02	0.98	1.06
	Sex x Cognitive Empathy	0.02 (0.03)	1.02	0.96	1.08
	Sex x Moral Disengagement	0.003 (0.006)	1.00	0.99	1.02
	Sex x Antisocial Behaviors	0.19 (0.16)	1.20	0.89	1.63
	Sex x Low perceived parental support	−0.003 (0.03)	0.99	0.94	1.06
	Sex x Low online parental monitoring	−0.04 (0.05)	0.96	0.87	1.05
	Sex x Aggression towards teachers	0.46 (0.12)	1.56 *	1.11	2.23
	Sex x Low perceived peer support	−0.03 (0.04)	0.97	0.91	1.04
	Sex x Low perceived significant other support	−0.002 (0.04)	0.99	0.92	1.08

Notes. *χ*^2^(57) = 1071.83, *p* < 0.001, *R*^2^ = 0.21 (Cox and Snell). 0.24 (Nagelkerke). * *p* < 0.05, ** *p* < 0.01, *** *p* < 0.001.

## Data Availability

Dataset is openly available at https://osf.io/uqcxs/overview?view_only=b544da9d547845a59ef1b3ff3b3047cf.
